# Aptamer-based Targeted Delivery of miRNA let-7d to Gastric Cancer Cells as a Novel Anti-Tumor Therapeutic Agent

**Published:** 2018

**Authors:** Puyan Daei, Mahsa Ramezanpour, Korosh Khanaki, Maryam Tabarzad, Iraj Nikokar, Mojtaba Hedayati CH, Ali Elmi

**Affiliations:** a *Medical Biotechnology Research Center, School of Paramedicine, Guilan University of Medical Sciences, Rasht, Iran. *; b *Protein Technology Research Center, Shahid Beheshti University of Medical Sciences, Tehran, Iran *; c *Department of Medical Microbiology, Faculty of Medicine, Guilan University of Medical Sciences, Rasht, Iran.*

**Keywords:** Nucleolin specific aptamer (aptNCL), miRNA let-7d, Gastric cancer, Conjugate, Targeted delivery

## Abstract

miRNAs as one of the potential therapeutic agents have been recently considered for cancer treatment. AS1411 (aptNCL) is a DNA aptamer specifically binding to nucleolin protein on the cancer cell surface with antiproliferative effect. The aim of the study was to develop a conjugate consisting of aptNCL (as targeted delivery of therapeutic agent) and miRNA let-7d (as a tumor suppressor) using two different linking methods and also to evaluate the potential effect of the conjugates on the proliferation of gastric cancer (MKN-45) cell line compared to negative control cell line of human dermal fibroblast (HDF). Conjugation was performed covalently by SM (PEG)_2_ as a bifunctional crosslinker (conjugate-1) and noncovalently, using 19bp complementary sticky end sequences (conjugate-2). Nucleolin positive MKN-45 and nucleolin negative HDF cells were cultured and treated with the conjugates. Then, the changes in let-7d expression and cell proliferation were determined using Real-time PCR and MTT methods, respectively. In MKN-45 cells, the conjugates caused significant increase in let7-d uptake compared with HDF cells (P = 0.0001). The conjugate-1, likely due to its higher stability compared with the conjugate-2, led to significantly more increase in intracellular let-7d in MKN-45 cells (30 fold versus 15 fold, respectively, P = 0.0001). The conjugates revealed more potent antiproliferative effect against gastric cancer cells compared with aptNCL alone (P = 0.0001). It was found that the aptNCL-let-7d conjugate efficiently carried let-7d into the cancer cells. Also, it appears that in the setting of aptNCL-let-7d conjugate, let-7d and aptNCL moieties could cooperate and synergistically exhibit the antiproliferative effect on cancer cells.

## Introduction

MicroRNAs (miRNAs) are endogenous short (about 19-23 nucleotides) double stranded non-coding RNA sequences that could control gene expression post- transcriptionally through stimulating mRNA degradation or preventing its translation (1, 2). Depending on the nature of the target genes, the miRNAs might be tumor suppressors, or oncogenes or both (3). Abnormality in miRNAs expression has been shown in various cancers containing gastric cancer (4, 5). Therefore, miRNAs as one of the potential therapeutic agents or targets have been recently considered for cancer treatment; however, their targeted delivery system is still a great challenge (6).

The human lethal-7 (let-7) family members have been reported as tumor suppressors with decreased expression level in several cancers like gastric (7), colon (8); however, there are exceptions in some cases (9). It has been revealed that restoration of let-7 miRNA expression level in cancer cells reduced tumorigenesis (10). It has been reported that let-7 family members by targeting oncogenes such as RAS (11), c-MYC (12), and STAT3 (13) might prevent cancer progression. Although let-7d has been less studied than other family members, its down-regulation in some cancers has been observed (14-16). 

Nucleic acid aptamers, also known as chemical antibodies, are small single stranded oligonucleotides usually about 15 to 70 nucleotides which could act as suitable tools for selective targeting cancer specific antigens with high affinity and specificity (17). Several aptamers have been developed against cancer cell surface antigens like prostate specific membrane antigen (PSMA-aptamer), nucleolin (AS1411-aptNCL), and Axl (GL21.T aptamer). In addition, aptamers can be combined with therapeutic agents such as nano- particles, or biological nano-molecules like miRNAs and consequently, can act as targeted drug delivery system (18). In the recent years, conjugating aptamer and miRNA as a chimeric therapeutic molecule has gained great attention. 

AS1411 (aptNCL) is a 26 mer G-rich DNA aptamer that specifically binds to nucleolin protein (19). It has been documented that nucleolin as a nuclear protein, is going to be overexpressed from the cell surface and cytoplasm of various kinds of cancers such as gastric cancer, however, not found on the surface of most natural cells (20-22). In this regard, the antiproliferative impact of AS1411 on cancers has been revealed (19). In addition, it can drive various linked nano- molecules particularly to the cancer tissues and moreover, into tumor cells cytoplasm, so, the promising property of AS1411 as a targeted delivery system for therapeutic agent has been reported (23, 24). 

In this study, a nano-molecule consisting of aptNCL and miRNA let-7d was designed to be conjugated. The conjugation was done using two different linkers, one by using the SM (PEG)_2_ hetero-bifunctional crosslinker and the other by using sticky end hybridization. The aim of the study was to specifically deliver let-7d to gastric cancer cells and also to evaluate the potential effect of these chimeras on the proliferation of gastric cancer (MKN-45) cell line compared to the negative control cell line of human dermal fibroblast (HDF).

## Experimental

*Cell culture*

MKN-45 (nucleolin positive) and HDF (nucleolin negative) cell lines were initially obtained from Pasteur Institute (Tehran, Iran). MKN-45 cells and HDF cells were grown in RPMI-1640 medium and Dulbecco’s modiﬁed Eagle’s medium (DMEM/F12), respectively at 37 °C in humidified air containing 5% CO_2_. The culture media were supplemented with 10% fetal bovine serum (FBS), 100 IU/mL penicillin, and 100 µg/mL streptomycin. The cell culture materials mentioned above were acquired from Gibco Company (Karlsruhe, Germany).

*Oligonucleotides* *and chemicals*

All of the oligonucleotides were synthesized by Bioneer (Korea). The sequences were as follows: 3′-thiolated AS1411** (**aptNCL): 5ʹ -GGT GGT GGT GGT TGT GGT GGT GGT GGA AAA A- 3ʹ (SH). Control aptamer: 5ʹ- GTA CAT TCT AGA TAG CC -3ʹ. miRNA let-7d 5p: 5ʹ- AGA GGU AGU AGG UUG CAU AGU U- 3ʹ. 3ʹ aminated miRNA let-7d 3p: 5ʹ -CUA UAC GAC CUG CUG CCU UUC U- 3ʹ (NH2). AS1411-stick: 5ʹ -GGT GGT GGT GGT TGT GGT GGT GGT GGA AGG CTA TCT AGA ATG TAC- 3ʹ. miRNA let-7d 3p-stick: 5ʹ -CUA UAC GAC CUG CUG CCU UUC UGU ACA UUC UAG AUA GCC UU- 3ʹ and miRNA mimic negative control (#SMC-2001, Bioneer, Korea).

According to the company guidelines, thiolated sequences were initially subjected to the reduction of SH terminal group and then immediately used in the following reactions. In the primary step, aptamer sequences were exposed to a short denaturation–renaturation stage to form G-quartet structure, as they were heated at 88 °C for 10 min and then cooled to room temperature (RT) for 15 min (22). 

Chemicals were PEGylated SMCC crosslinker (Thermo Fisher Scientific, USA), Dithiothreitol (DTT) (Sigma, USA), Lipofectamine 2000 (Sigma, Germany), DMSO (Gibco, Germany), Acrylamide (Sumchun, China), TEMED (Bio Rad, Germany), and DNA ladder 10bp (GeneOn, Germany). 

The aptNCL-miRNA conjugate was consisted of two entities, aptNCL and miRNA let-7d, which was linked together by two different methods:

*Preparation of aptNCL -miRNA let-7d conjugate using SM (PEG)*_2_ *cross linker*

As the first step, disulfide bonds in Thiol-modified aptNCL were reduced to SH group (SH-apt). Briefly, 100 µL of thiolated aptamer solution (6 nmol) was mixed with 100 µL of 1M DTT solution (pH 6) and incubated at RT for 45 min followed by 37 °C for 15 min. To eliminate excess DTT, the solution was extracted three times by the same volume of Ethyl acetate. 

At the second step, the amine-modified miRNA let-7d duplex was incubated with a 50-fold molar excess of SM (PEG)_2_ in PBS (pH 7.2) containing 3mM EDTA as the conjugation buffer at 37 °C for 1 h, then at 4 °C overnight. Excess SM (PEG) _2_ was removed using Amicon Ultra 0.5 mL Centrifugal Filter (cut off: 3kD; Merck Millipore, Germany). The sample was centrifuged and equilibrated with the conjugation buffer. Finally, the column was reversed and centrifuged to collect the purified sample. At the last step, purified miRNA let-7d-SM (PEG)_2_ and aptNCL-SH were mixed together in equal molar ratio at RT for 1 h, then an overnight at 4 °C. The conjugate formation was confirmed by poly acrylamide gel electrophoresis (PAGE, 15%) and the construct stability was evaluated in human serum. 

*Preparation of aptNCL -miRNA let-7d conjugate using sticky ends*

Equimolar ratio of aptamer-stick, miRNA let-7d 5p, and miRNA let-7d 3p-stick were separately dissolved in conjugation buffer (pH 7.4) and heated at 95 °C for 2 min, then cooled at 37°C for 15 min. Then, immediately were added to each other and incubated at 30 °C for 30 min followed by an adjustment step at RT for 30 min. The conjugation efficacy was assessed by PAGE 15%, and the stability of the conjugate was examined in human serum.

*Evaluation of aptNCL -miRNA let-7d stability in human serum*

To evaluate the serum stability of both conjugates, 4 µM of the conjugates were subjected to 80% human serum (Type AB^+^) from 1 to 48 h. At the indicated time intervals (0, 1, 2, 4, 6, 12, 24 and 48h), 8 µL (32 pmol) of each sample was extracted and incubated at 37 °C for 2 h with 1 µL proteinase K solution (600 mAU/mL) (Sigma, Germany) to eliminate serum proteins interference in electrophoresis. Then, 9 µL from 1X Tris*-*Borate*-*EDTA (TBE) and 3 µL of gel loading buffer (GeneOn, Germany) were added and kept at -80 °C. Finally, all samples were analyzed by 15% PAGE. The gel was stained by SYBR Green DNA safe stain (Sinaclone, Iran) (25).

*Cell proliferation assay*

Cell proliferation was assessed by using 3-(4, 5-Dimethylthiazol-2-yl)-2, 5-diphenyltetrazolium bromide (MTT) assay kit (BioBasic, Canada). Briefly, MKN-45 and HDF cells were seeded at 7,000 cells per well (96-well plates) in their respective media. After 24 h of seeding, and following 4 h of starvation using the serum-free media, the cells were treated with the conjugates , aptNCL, control aptamer, mixture of aptNCL plus miRNA let-7d, or transfected with miRNA let-7d , miRNA mimic negative control for 8h and incubated for 24 and 48 h before MTT assay, separately. MTT solution (100 µL/well, 5 mg/mL) was added to each well and then incubated for 4 h at 37 °C. After removing the medium, 100 µL of DMSO was added in each well and the plate was shaken for 10 min at 100 rpm for dissolving the formazan crystals. The absorbance of each well was measured by a microplate reader (Stat Fax-2100, Awareness, USA). The experiments were done in triplicate. The results of each treatment were normalized with the control medium and relative proliferation activity was determined as a percentage of the control (activity of control was considered as 100). miRNA let-7d and miRNA mimic negative control were transfected using Lipofectamine 2000 according to the manufacturer’s protocol. The half maximal inhibitory concentration (IC50) of the conjugate-1 and conjugate-2 was determined for 48 h (26). 

*miRNA let-7d quantification using quantitative RT-PCR*

MKN-45 and HDF cells were seeded at 40,000 cells per well (24-well plates) in their relevant media. After 24 h of seeding, and following 4 h of starvation using the serum-free media, the cells were treated with the conjugates (at IC50 concentrations), aptNCL, control aptamer, and mixture of aptNCL plus miRNA let-7d (200nM) or transfected with let-7d miRNA and control-miRNA (200nM) and incubated for 24 and 48 h, separately. In two time point of 24 h and 48h, the relative expression of miRNA let-7d was detected by quantitative RT-PCR technique. Briefly, RNAs were recovered by 1mL of RiboEx RNA extraction kit (Gene all, Korea) according to the manufacturer’s instructions. To evaluate the intracellular levels of the let-7d miRNA (target gene) or U6 RNA (housekeeping control gene) (Cat #MS00033740, Qiagen, Germany), 500 ng of total RNA was reverse transcribed by using miScript II Reverse Transcription cDNA synthesis kit (Qiagen, Germany) in a final volume of 20 µL consistent with manufacturer’s protocol: 60 min 37 °C then 5 min 95 °C by Mastercycler gradient (Eppendorf, Germany), then, amplification was done using the miScript-SYBR Green PCR Kit and speciﬁc miScript Primer Assay (Cat #MS00003136, Qiagen, Germany) and analyzed using the StepOne^TM^ cycler real-time PCR system (Applied Biosystems, USA). The ΔΔCT method was used for relative quantization of miRNA levels. Each sample was run in triplicate.

*Statistical Analysis*

Data are presented as mean ± SD. Inter-group comparisons were performed us­ing the one-way analysis of variance (ANOVA) followed by post hoc Tukey’s test. In each group, Paired t-test was used to compare between 24h and 48 h. For all tests, *P* < 0.05 was considered as statistically significant. The data were analyzed using SPSS software version 22.

## Results

*Preparation of aptNCL - miRNA let-7d conjugates*

**Figure 1:**
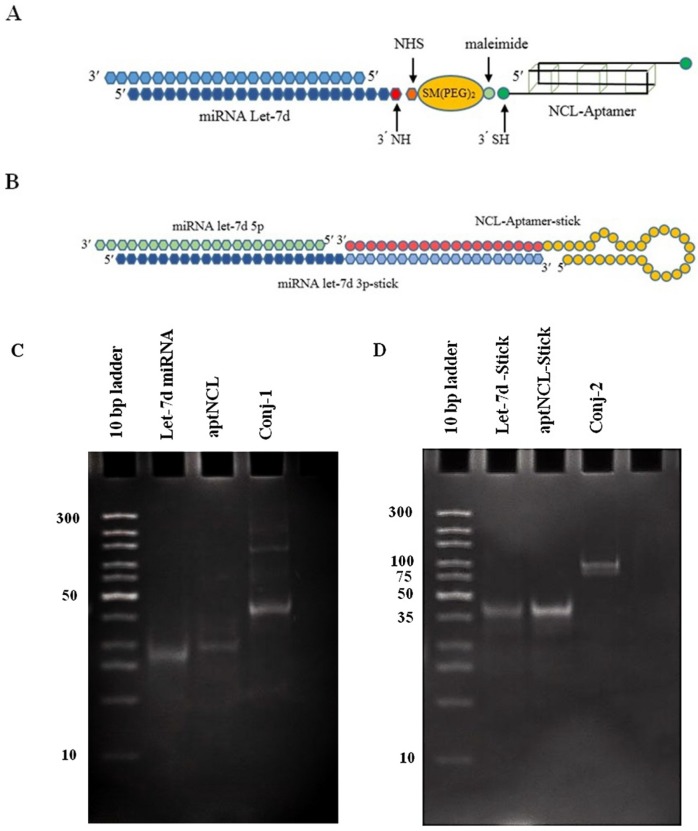
AptNCL-let-7d conjugates design and formation. (A) Schematic illustration of aptNCL-let-7d conjugate using covalent SM (PEG)_2 _crosslinker (conjugate-1). (B) Schematic illustration of annealed aptNCL-let-7d conjugate using non-covalent sticky-end hybridization (conjugate-2). To confirm the formation of the conjugates, (C) let-7d, aptNCL, the conjugate-1 (D) let-7d-stick, aptNCL-stick and the conjugate-2 were assessed on 15% non-denaturing polyacrylamide gel and stained by SYBR Green DNA safe stain.

As mentioned earlier, the aptNCL-miRNA conjugate contained two entities, aptNCL and miRNA let-7d, linked together by two methods:

In the first method, SM (PEG)_ 2_, a heterobifunctional crosslinker was used. Modified oligonucleotides were conjugated through two sequential reaction steps. The NHS end of the SMCC reacted with the primary amine at the 3′ end of miRNA sequence in the first step, through the amide bond. The reaction efficiency depends substantially on the pH of the conjugation buffer which should be carefully controlled (optimum pH 7-9) to prevent undesired reaction of the amine group with the maleimide fragment of the SM(PEG)_2_ crosslinker (27). At the second step, the aptNCL sequence containing the thiol group reacted with the maleimide functional fragment in the SM (PEG)_2_ which optimized reaction was performed in mildly acidic to neutral condition (pH 6.5-7.5). In this study, optimal pH was considered 7.2 for both reactions of the SM (PEG)_2_ functional groups (Figure 1A). Considering the size of miRNA sequence (21bp) and aptNCL (31bp), it was expected that the final product to be appeared around 52 bp following 15% PAGE. The successful formation of the final product was visible as a specific band around 50 bp (Figure 1C). A slight change from the exact length of the oligonucleotide could be due to the secondary structures formed in the aptamer part of the conjugate, as well as the heterogeneity of the conjugate composition as one DNA part and an RNA part. Besides, several other weak bonds observed in upper molecular weights might probably be the result of unspecific binding of other amine groups in RNA/DNA structure to the linker, which led to the formation of conjugates containing multiple copy of the aptamer sequence. However, the main product of the reaction (presented as the intense bond on gel electrophoresis) was the favor conjugate. 

In the second method, a 19bp sticky part was used as a linker for conjugation. The aptNCL was synthesized as attached to a 19bp sticky sequence which was complementary to the sticky sequence linked to 3′ end of let-7d-3p (Figure 1B). Considering the size of stick-let-7d-3p strand (41bp) and the aptNCL-stick sequence (45 bp), the conjugate was expected to visualize around 86bp. Regarding the results of the electrophoresis, the accuracy of the conjugate was confirmed by observing the band in the range of 75-100bp (Figure 1D).

*Stability of aptNCL- miRNA let-7d conjugates in human serum*

**Figure 2:**
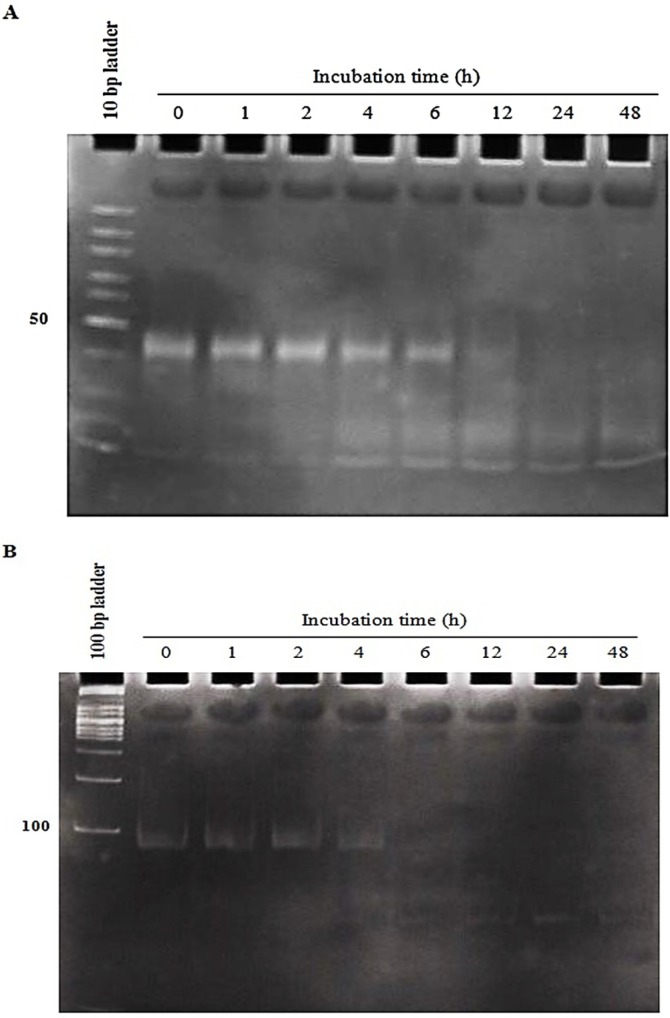
The conjugates stability in human serum for the specified incubation times. aptNCL-let-7d conjugates were incubated in the 80% human serum for 0-48 h at 37  C and then miRNA degradation and loss of accurate folding at the indicated time intervals was assessed through electrophoresis on 15% non-denaturing polyacrylamide gel stained with SYBR Green DNA safe stain

As shown in Figure 2, the two conjugates illustrated different stability in human AB+ serum. The conjugate-1 (using covalent SM (PEG)_ 2 _crosslinker) was more stable than the conjugate-2 (using non-covalent sticky-end hybridization) (at least 6 h versus 4 h, respectively) (Figure 2A and Figure 2B). After the above mentioned time points, the conjugates gradually started to be degraded and wiped out.

*Selective antiproliferative effect of aptNCL - miRNA let-7d conjugates on MKN-45 cells*

**Figure 3:**
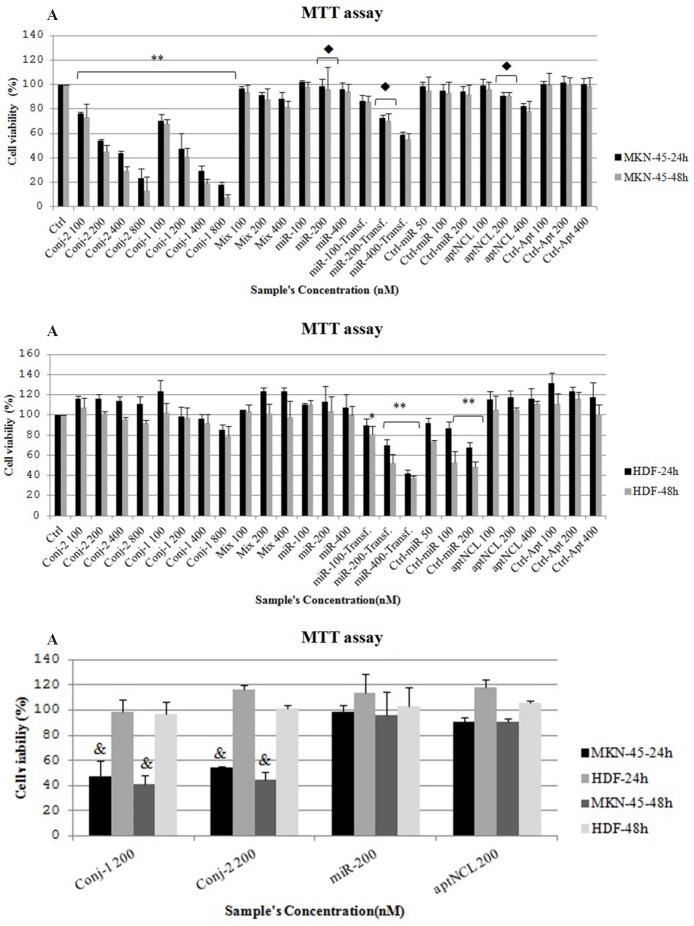
Proliferation activity in human gastric cancer (MKN-45) cell line (A) and human dermal fibroblast (HDF) cell line (B) treated with the conjugates (100, 200, 400 and 800 nM), aptNCL, control aptamer, mixture of aptNCL plus miRNA let-7d (100, 200 and 400 nM ) or transfected with miRNA let-7d (100, 200 and 400 nM) and miRNA mimic negative control(50, 100 and 200nM) after 24 h and 48 h. (C) Comparison of cell proliferation between MKN-45 and HDF cell lines under similar treatments. ** *P *<0.0001 and * *P* < 0.05 by comparison with the untreated control cells;   *P *< 0.0001 by comparison with the conjugate-1 and conjugate-2; ^&^*P* <0.0001 by comparison with HDF cells at the matched times. Data are presented as mean ± SD. Ctrl, Control; Conj, Conjugate; Transf, Transfected; aptNCL, nucleolin aptamer

In MKN-45 cells, the conjugates led to inhibition of cell proliferation in a time and dose-dependent manner (Figure 3A). The conjugates revealed more potent antiproliferative effect against MKN-45 cells compared to aptNCL alone after 24 and 48 h, separately (*P *= 0.0001) (Figure 3A). IC_50_ for conjugate-1 and conjugate-2 was 200 nM and 250nM, respectively. Whereas, transfected miRNA let-7d caused significantly more inhibition on the cell proliferation of MKN-45 cancer cells as compared with the conjugates (*P *= 0.0001) (Figure 3A). The proliferation activity in MKN-45 cell line was significantly lower in 48h than 24h under the conjugate-2 treatment (*P *= 0.0001) (Figure 3A).

The proliferation activity of HDF cell line was similar under untreated control condition and under each treatment with the conjugates after 24 and 48 h, separately (Figure 3B), high concentration of the conjugate-1 and conjugate-2 (800nM) inhibited the proliferation of HDF cells by about 10% and 20%, respectively, although, there were not statistically significant (Figure 3B). But, compared to the conjugates, the transfected let-7d miRNA significantly decreased the proliferation of the HDF cells which could be partly attributed to the effect of Lipofectamine (*P* = 0.0001) (Figure 3B). This finding confirmed the unspecific side effects of let-7d on normal cells when was delivered through non-targeted strategy. In contrast, the proliferation of the HDF cells was not significantly different between 24 h and 48 h under each of the matched conjugate treatments (Figure 3B).

In MKN-45 cells compared to HDF cells, the conjugates led to significant inhibition of cell proliferation after both 24 h and 48 h (*P *= 0.0001) (Figure 3C). Also, aptNCL alone showed remarkably antiproliferative effect on MKN-45 cells compared to HDF cells after both 24 h and 48 h (*P* < 0.05) (Figure 3.C).

*aptNCL - miRNA let-7d conjugates increased miRNA let-7d level in MKN-45 cells*

Previous studies have demonstrated that the aptNCL can be bound and internalized in NCL-expressing cells (28, 29). MKN-45 and HDF cells were treated with 200nM conjugate-1, 250nM conjugate-2, 200nM mixture of aptNCL and miRNA let-7d and control-oligonucleotides or transfected with 200nM let-7d miRNA and control-miRNA for 24 h and 48 h. 

**Figure 4:**
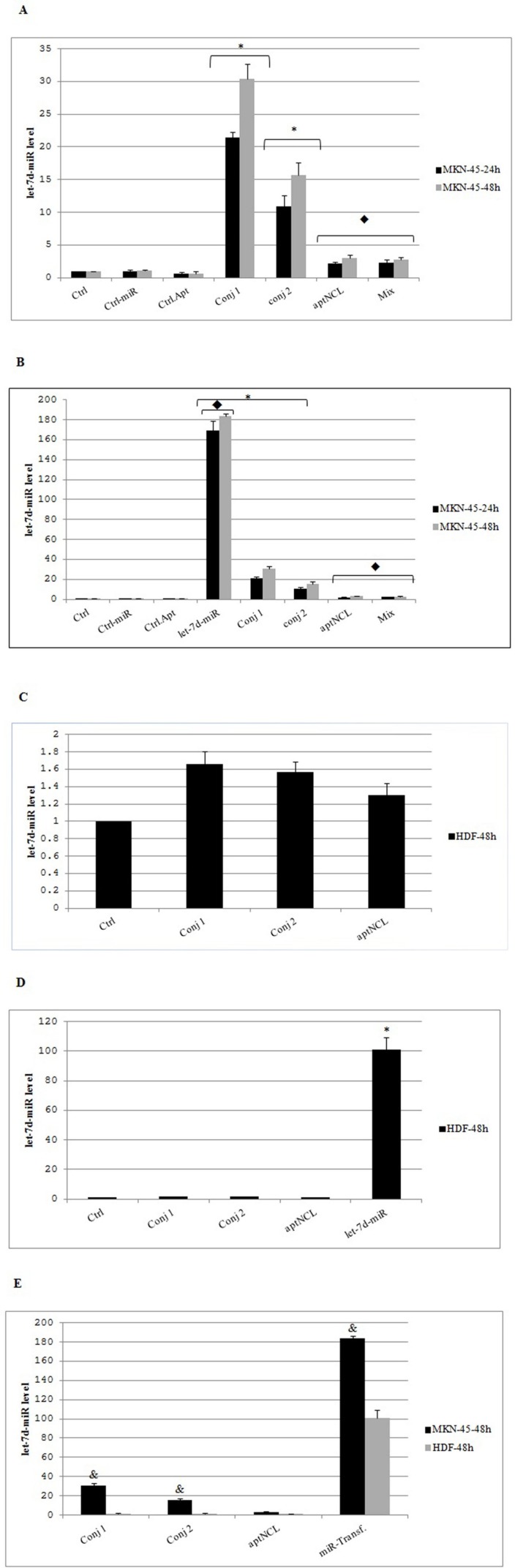
Relative expression of intracellular miRNA let-7d in different experimental groups. (A) MKN-45 cells (NCL^+^) were treated with 200nM conjugate-1, 250nM conjugate-2, 200 nM mixture of aptNCL plus miRNA let-7d and control-oligonucleotides or (B) transfected with 200 nM miRNA let-7d and control-miRNA. After 24 and 48 h, relative miRNA let-7d levels were quantified by qRT-PCR. (C) HDF cells (NCL^-^) were treated or (D) transfected with the indicated concentrations of the oligonucleotides. After 48 h, relative miRNA let-7d levels were assessed by qRT-PCR. (E) Comparison of intracellular let7d between MKN-45 and HDF cell lines under similar treatments. * *P* < 0.0001 by comparison with the untreated control cells;   *P *< 0.0001 by comparison with the conjugate-1 and conjugate-2; ^&^*P *< 0.0001 by comparison with HDF cells. Data are presented as mean ± SD. Ctrl, Control; Conj, Conjugate; Transf, Transfected; aptNCL, nucleolin aptamer

In MKN-45 cells (NCL^+^), the conjugates compared to control condition resulted in significant increase in intracellular level of let-7d in a time-dependent manner (24 h to 48 h). Also, in these cancer cells, the conjugate-1, likely due to its higher stability compared with the conjugate-2, led to significantly more increase in intracellular let-7d compared with the conjugate-2 (30 fold versus 15 fold, respectively, *P *= 0.0001) (Figure 4A). 

The let-7d level in HDF cells (NCL^-^) was unchanged under each of the conjugate treatments compared to the untreated control condition (Figure 4C). These data confirmed the low expression level of NCL on HDF cells surface, and also could be an indication of receptor-dependent delivery and intracellular processing of the let-7d miRNA entity of the conjugates in MKN-45 cancer cells.

In MKN-45 cells, treatment with a mixture of aptNCL and miRNA let-7d did not affect the level of intracellular let-7d compared with untreated control condition, confirming that the entry of the conjugate into the cancer cells depended on the presence of the aptamer entity within the conjugate that delivered miRNA part inside the cells. 

On the other hand, partly similar to MKN-45 cells, compared to the conjugates, the transfected miRNA let-7d significantly increased the expression level of the let-7d in HDF cells (*P* = 0.0001) (Figure 4.B and D). This result reveals the similar non-specific impacts of lipid carrier in the entry of let-7d into the both cancer and normal cells. Consequently, because of the lack of an aptamer-mediated targeted delivery system, the side effects of the lipid carrier in the normal cells might occur.

In MKN-45 cells compared with HDF cells, the conjugates showed significant increase in intracellular level of let-7d after 48 h (*P *= 0.0001) (Figure 4E). Also, the transfected miRNA let-7d resulted in significantly more increase in the expression level of the let-7d in MKN-45 cells compared to HDF cells after 48 h (*P *= 0.0001) (Figure 4.E).

The let-7d level in HDF cells (NCL^-^) was unchanged under each of the conjugate treatments compared to the untreated control condition (Figure 4C). These data confirmed the low expression level of NCL on HDF cells surface, and also could be an indication of receptor-dependent delivery and intracellular processing of the let-7d miRNA entity of the conjugates in MKN-45 cancer cells.

In MKN-45 cells, treatment with a mixture of aptNCL and miRNA let-7d did not affect the level of intracellular let-7d compared with untreated control condition, confirming that the entry of the conjugate into the cancer cells depended on the presence of the aptamer entity within the conjugate that delivered miRNA part inside the cells. 

On the other hand, partly similar to MKN-45 cells, compared to the conjugates, the transfected miRNA let-7d significantly increased the expression level of the let-7d in HDF cells (*P* = 0.0001) (Figure 4.B and D). This result reveals the similar non-specific impacts of lipid carrier in the entry of let-7d into the both cancer and normal cells. Consequently, because of the lack of an aptamer-mediated targeted delivery system, the side effects of the lipid carrier in the normal cells might occur.

In MKN-45 cells compared with HDF cells, the conjugates showed significant increase in intracellular level of let-7d after 48 h (*P *= 0.0001) (Figure 4E). Also, the transfected miRNA let-7d resulted in significantly more increase in the expression level of the let-7d in MKN-45 cells compared to HDF cells after 48 h (*P *= 0.0001) (Figure 4.E).

## Discussion

Nowadays, the restoration of miRNAs in cancer cells has been considered as a new therapeutic approach. Nevertheless, there are some challenges in the application of miRNAs in clinic, including low systemic stability because of the degradation by endonucleases/exonucleases, rapid renal elimination, and lack of efficient delivery to the cancer cells due to their negative charge (30). Aptamers are an emerging class of the molecules that can be used as drug targeting carriers, in addition to their diagnostic and therapeutic roles (18, 31). In the present study, we successfully designed an efficient targeted oligonucleotide delivery system containing two moieties, AS1411 (aptNCL), a DNA aptamer that could bind to the nucleolin receptor (apt-NCL) and miRNA let-7d as a pharmacological agent, using two different linking methods. 

In the first method, SM (PEG) _2_, a chemical linker was used to create the desired conjugate. SMCC is known to be one of the most commonly bifunctional crosslinkers, one of which used in this study has two polyethylene glycol as the spacer [SM (PEG)_ 2_] to provide an appropriate space between two oligonucleotides for their proper functionality, as the main challenge in the design of this construct was the interference between the miRNA molecule and the aptamer sequence which might result in the loss of the functionality of both moieties. Due to the use of PEG, increased solubility, reduced accumulation, reduced immunogenic responses, and increased flexibility of the conjugate could be 

expected (32). 

Although several studies have been carried out using the SMCC linker and similar bifunctional crosslinkers to bind protein molecules (33, 34), but to date, few studies have directly conjugated two oligonucleotide sequences using this type of linker. As an example, in a study conducted by Zhao *et al.,* the A10-3.2 aptamer, a specific prostate-specific membrane antigen (PSMA) antagonist, was linked to two different miRNAs (miR-15a and miR-16-1) using the SMCC and its therapeutic effect on prostate metastatic cancer cells was analyzed. The results confirmed the targeted delivery of miRNAs to the prostate cancer cells and introduced SMCC as an effective method for the conjugation of oligonucleotide sequences (35). In the study of Lai *et al.*, SMPB was used as a hetero-bifunctional cross linker with a similar mechanism of SMCC to develop aptNCL-siRNA chimeras. Biological activity confirmed the cell selectivity of the resulted chimeras towards lung adenocarcinoma (CL1-5) cell line with the great level of nucleolin expression compared to the control cell line of Human Umbilical Vein Endothelial Cells (HUVEC) with poor expression of nucleolin (27). In our study, similar results were observed, so that, following cell treatment with the optimized aptNCL-let-7d conjugate, its uptake into the nucleolin positive cancer cells (MKN-45) was remarkably greater than nucleolin negative cells (HDF). Also, the conjugate of aptNCL-let-7d exhibited more activity against proliferation of gastric cancer cells compared with aptNCL alone.

In the second linking strategy, a 19bp complementary sticky sequence was used as the linker and spacer. By using this non-covalent method, the interference between two oligonucleotide sequences was prevented by inserting 19bp distance between two parts. In contrast to the SMCC, several studies have been done using this method. Esposito *et al.* introduced the conjugate of the let-7g passenger strand to the 3′ end of the GL21.T aptamer (tyrosine kinase receptor ligand) by using 17bp stick-base, and then, by annealing of the complementary guide strand of the miRNA, showed an efficient delivery of let-7g to target cancer cells (26). In another study in 2015, using the stick-base non-covalent technique, the conjugate based on the GL21.T aptamer and antimiR-222 was developed (25). In the study of Catuogno *et al*., the stability of the conjugate (GL21.T aptamer and antimiR-222) was longer than our conjugate (aptNCL-let-7d) (25). In addition to the dissimilarity in oligonucleotide sequences (aptamer or miRNA), this difference in the stability may be partly attributed to the stabilizing chemical modification in the nucleotides of miRNA sequences, as 2′-F-pyrimidines used in the let-7g study (26). This chemical modification increases the stability of the oligonucleotides against serum nuclease; however, it should not be ignored that less modification in the miRNA sequence can help to better identification by the RNA-induced silencing complex (RISC) complex (36, 37). In this study, it was found that the conjugate-1 was more stable than the conjugate -2 likely due to the strong covalent bonds by SM (PEG)_ 2_ versus the hydrogen bonds by complementary stick-base, respectively. Also, likely because of the more stability of the conjugate-1 compared with the conjugate-2; intracellular levels of let-7d in MKN-45 cells were significantly increased.

As the conclusion of study by Esposito *et al*., it was stated that the effect of the conjugate on cancer cell proliferation seems to be additive and could be attributed to the combined influences of GL21.T aptamer and miRNA let-7g on Axl signaling pathway and on target genes of let7g, respectively (26). In the present study, it seemed that in the setting of aptNCL-let-7d conjugate, let-7d and aptNCL moieties could cooperate and synergistically exhibit the antiproliferative effect on gastric cancer cells. 

## Conclusion

In summary, this study addressed the development of a novel combined therapeutic strategy composed of AS1411 aptamer )aptNCL( and let-7d miRNA that can be used to target the gastric cancer cells with overexpression of nucleolin on their cell surface. It was also demonstrated that the overexpression of the let-7d in gastric cancer cells by the specific aptamer-mediated delivery system synergistically reduced the cell proliferation. More research exploring the therapeutic efficacy of the conjugate against other nucleolin positive cancer cell lines as well as its *in-vivo* activity would be welcomed.
